# CHANGES OVER TIME IN WORKING LIFE EXPECTANCY AT OLDER AGES IN SINGAPORE: A COMPARISON OF TWO COHORTS

**DOI:** 10.1093/geroni/igae098.1420

**Published:** 2024-12-31

**Authors:** Rahul Malhotra, Abhijit Visaria, Angelique Chan, Stefan Ma, Yasuhiko Saito

**Affiliations:** Duke-NUS Medical School, Singapore, Singapore; Duke-NUS Medical School, Singapore, Singapore; Duke-NUS Medical School, Singapore, Singapore; Ministry of Health Singapore, Singapore, Singapore; Nihon University, Tokyo, Japan

## Abstract

Singapore, a rapidly ageing country, underwent significant development in a short time after independence in 1965. We study whether differences based on educational attainment in the average years of remaining life expected to be spent working (i.e., working life expectancy), have changed among older persons over time. We use data from two representative studies of older persons: initiated in (i) 2009 (n=4990) with follow-ups in 2011-12, 2015, and (ii) 2016-2017 (n=4549) with follow-up in 2019. We estimated working (part- or full-time current employment) life expectancy using the multistate life table method. We included sex (male / female) and education (lower [no formal or primary] / higher [secondary and above]) as covariates and controlled for ethnicity and time-varying functional health. In the earlier cohort, working life expectancy is estimated higher among those with higher education (e.g. at age 60: 9.2 years [95% CI: 8.0-10.4] among males and 6.5 [6.3-6.6] years among females) versus those with lower education (7.6 [6.9-8.4] years among males and 5.4 [5.2-5.7] years among females). This educational difference reverses in the more recent cohort: working life expectancy is higher among those with lower education (at age 60: 10.0 [8.4-11.7] years among males and 7.7 [6.4-9.0] years among females) versus those with higher education (8.9 [7.6-10.3] among males and 6.5 [5.2-7.7] among females). This change was driven by an increase in working life expectancy among those with lower education and no change among the higher educated. This increase suggests both more employment opportunities and the requirement for continuing employment.

